# Properties and Crystallization Phenomena in Li_2_Si_2_O_5_–Ca_5_(PO_4_)_3_F and Li_2_Si_2_O_5_–Sr_5_(PO_4_)_3_F Glass–Ceramics Via Twofold Internal Crystallization

**DOI:** 10.3389/fbioe.2015.00122

**Published:** 2015-09-03

**Authors:** Markus Rampf, Marc Dittmer, Christian Ritzberger, Marcel Schweiger, Wolfram Höland

**Affiliations:** ^1^Research and Development, Inorganic Chemistry, Technical Fundamentals, Ivoclar Vivadent AG, Schaan, Liechtenstein

**Keywords:** glass–ceramics, lithium disilicate, calcium fluoroapatite, strontium fluoroapatite, prosthodontics, twofold crystallization

## Abstract

The combination of specific mechanical, esthetic, and chemical properties is decisive for the application of materials in prosthodontics. Controlled twofold crystallization provides a powerful tool to produce special property combinations for glass–ceramic materials. The present study outlines the potential of precipitating Ca_5_(PO_4_)_3_F as well as Sr_5_(PO_4_)_3_F as minor crystal phases in Li_2_Si_2_O_5_ glass–ceramics. Base glasses with different contents of CaO/SrO, P_2_O_5_, and F^−^ were prepared within the glasses of the SiO_2_–Li_2_O–K_2_O–CaO/SrO–Al_2_O_3_–P_2_O_5_–F system. Preliminary studies of nucleation by means of XRD and scanning electron microscopy (SEM) of the nucleated base glasses revealed X-ray amorphous phase separation phenomena. Qualitative and quantitative crystal phase analyses after crystallization were conducted using XRD in combination with Rietveld refinement. As a main result, a direct proportional relationship between the content of apatite-forming components in the base glasses and the content of apatite in the glass–ceramics was established. The microstructures of the glass–ceramics were investigated using SEM. Microstructural and mechanical properties were found to be dominated by Li_2_Si_2_O_5_ crystals and quite independent of the content of the apatite present in the glass–ceramics. Biaxial strengths of up to 540 MPa were detected. Ca_5_(PO_4_)_3_F and Sr_5_(PO_4_)_3_F influence the translucency of the glass–ceramics and, hence, help to precisely tailor the properties of Li_2_Si_2_O_5_ glass–ceramics. The authors conclude that the twofold crystallization of Li_2_Si_2_O_5_–Ca_5_(PO_4_)_3_F or Li_2_Si_2_O_5_–Sr_5_(PO_4_)_3_F glass–ceramics involves independent solid-state reactions, which can be controlled via the chemical composition of the base glasses. The influence of the minor apatite phase on the optical properties helps to achieve new combinations of features of the glass–ceramics and, hence, displays new potential for dental applications.

## Introduction

Glass–ceramics are the result of the controlled devitrification of base glasses including nucleation and crystallization and represent a distinct category of technical materials. The development of glass–ceramics targets the creation of specific material properties as well as property combinations. Wild crystallization is counterproductive and has to be avoided. The chemical composition of the base glass, nucleation techniques, and crystallization heat treatments, hence, are the most important parameters where research and development of these materials begins (Höland and Beall, 2012). In this manner, the knowledge about processes and mechanisms involved in glass–ceramic technology has been continuously advanced since in the mid 1950s Stookey (1959) discovered the first glass–ceramic material via precipitation of Li_2_Si_2_O_5_ from a base glass using Ag clusters as agent for heterogeneous nucleation. Li_2_Si_2_O_5_ is still subject of a multitude of glass–ceramic research. Nucleation mechanisms have been extensively studied (Headley and Loehman, 1984) and besides Stookey’s heterogeneous nucleation, Vogel (1963) found evidence for the influence of glass-in-glass phase separations on nucleation of lithium silicate glass–ceramics from non-stoichiometric base glasses. Enhancing its mechanical and chemical properties, since that time product-oriented research and development on Li_2_Si_2_O_5_ crystals focused on multi-component glass systems, including P_2_O_5_ as agent for internal heterogeneous nucleation.

Höland et al. (1994) developed translucent glass–ceramics for dental applications in the system SiO_2_–Li_2_O–ZrO_2_–P_2_O_5_ initiating further in depth studies of the glass system extended by, e.g., Al_2_O_3_ and K_2_O (Bischoff et al., 2011). Though, the final identification of the exact mechanisms running is difficult, basic principles of the crystallization and nucleation have been understood. Li_2_SiO_3_ and Li_2_Si_2_O_5_ crystals precipitate in a parallel reaction at temperatures <600°C before the crystallization rate of Li_2_Si_2_O_5_ overcomes the one of Li_2_SiO_3_ at temperatures >700°C. Dissolution of Li_2_SiO_3_ at higher temperatures initiates an enhanced secondary crystallization of Li_2_Si_2_O_5_. While amorphous lithium phosphate can be found in form of glass-in-glass phase separations in the nucleated base glass, crystalline Li_3_PO_4_ was only found after heat treatments >780°C (Höland and Beall, 2012). Amorphous or disordered nanoscale Li_3_PO_4_ phase separations play an uncontroversial role in the nucleation mechanism of lithium silicate. Based on the sound knowledge about nucleation and crystallization mechanisms, materials for restorative dentistry with a toughness of up to 2.9 MPa*m^0.5^ and flexural biaxial strength up to 600 MPa with well-suited optical properties could be achieved (Cramer von Clausbruch et al., 2000; Höland et al., 2005; Apel et al., 2007, 2008; Höland and Beall, 2012). Furthermore, Li_2_Si_2_O_5_ glass–ceramics are suited for various processing techniques, such as sintering, molding, and CAD/CAM machining in an intermediate Li_2_SiO_3_ stage of the glass–ceramic.

However, to meet the special demands for esthetics in prosthodontics, more and more advanced glass–ceramics are needed. Multi-component glass systems allowing the controlled precipitation of more than a single crystal phase in the glassy matrix display state of the art technique with high potential (Ritzberger et al., 2015). Already in 1994, an apatite-containing leucite glass–ceramic for use in restorative dentistry was reported by Höland et al. (1994, 2000). By applying a combination of surface nucleation during which leucite formed and controlled internal nucleation of fluoroapatite, Höland et al. (1994, 2000) managed to produce leucite–apatite glass–ceramics in the SiO_2_–Al_2_O_3_–CaO–Na_2_O–K_2_O–P_2_O_5_–F base glass system, which led to the development of the commercial product IPS d.Sign^®^. Contrary to numerous research published on the development of bioactive glass–ceramics for biomedical materials, in the field of prosthodontics the use of fluoroapatite is limited to the control of optical or mechanical material properties. The minor apatite phase in the leucite glass–ceramics introduced a special hue originating in the bulk of the material that helped to achieve controlled translucency comparable to natural teeth.

A first report on the parallel existence of Li_2_Si_2_O_5_ crystals and fluoroapatite in a devitrificateted glass was made by Kuzielova et al. (2006). However, a bioactive and, hence, chemically not durable glass was subject of these studies. The present work illustrates the qualitatively and quantitatively controlled internal crystallization of fluoroapatite within different chemically durable glass–ceramics (without adding a different material, such as a glass) with Li_2_Si_2_O_5_ as main crystal phase. Preliminary studies on phase separation processes occurring during the nucleation heat treatment and principles of twofold crystallization are shown. Furthermore mechanical and optical properties of the resulting glass–ceramics are discussed with respect to dental applications.

## Materials and Methods

### Glass formation and thermal analysis

Using the raw materials quartz, lithium carbonate, potassium carbonate, calcium carbonate or strontium carbonate, aluminum oxyhydroxyhydrate, aluminum metaphosphate and calcium fluoride or lithium fluoride base glasses of different compositions were melted as shown in Table 1. Melting took place in a Pt-Rh10 crucible at 1500°C in an electric furnace. After a dwell time of 1 h, the glasses were quenched in water. Thermal analysis of the dried glass granules measuring less than about 1 mm in diameter was performed in nitrogen atmosphere by differential scanning calorimetry (DSC) using a Netzsch STA 409 apparatus, regulated at a heating rate of 10 K/min. An accuracy of ±1°C can be reached according to the reference measurements using the transition of low-quartz to high-quartz at 573°C as a standard gage.

**Table 1:** **Chemical compositions of the base glasses calculated from the initial weight of the raw materials, taking into account the evaporation of P_2_O_5_ and F^−^**.

	A	B	C	D	E	F	G
mol% SiO_2_	65.0	63.9	62.8	62.4	59.7	63.5	62.8
mol% Li_2_O	27.0	26.6	26.2	26.0	24.8	26.5	26.2
mol% K_2_O	2.0	2.0	2.0	2.0	2.0	2.0	2.0
mol% CaO	3.0	4.0	5.0	5.4	8.0	5.0	–
mol% SrO	–	–	–	–	–	–	5.0
mol% Al_2_O_3_	1.5	1.5	1.5	1.5	1.5	1.5	1.5
mol% P_2_O_5_	0.9	1.2	1.5	1.6	2.4	1.5	1.5
mol% F^−^	0.6	0.8	1.0	1.1	1.6	^−^	1.0

In a subsequent step, the quenched glass frits were melted again at a temperature of 1500°C. After a dwell time of 2 h, the glasses were cast into a graphite mold to form blocks measuring about 13 mm × 14 mm × 30 mm. The glass blocks were immediately removed from the mold and cooled to room temperature under ambient conditions.

### Nucleation

Besides nucleation, the first heat treatment was required to relief stresses in the glass structure and, hence, allows further machining of the glass. The cooled glass blocks were placed in a furnace pre-heated to 500°C. After a dwell time of 30 min, the glass blocks were allowed to cool to room temperature. To study the effect of the dwell time at 500°C, one glass block of glass D (Table 1) was heat treated for 10 h prior to cooling. The cooling rate in the glass transition range was approximately 2–3 K/min. Small glass plates were cut from the glass blocks. For crystal phase analysis, the surface of the glass plates was ground using a 40-μm diamond grit grinding disk. X-ray diffraction patterns (XRD patterns) were recorded in a 2θ-range from 10° to 60° in 0.014° steps (D8 Advance, Bruker, Karlsruhe, Germany) using CuK_α_ radiation (λ = 0.154 nm). Micrographs of the glass plates after nucleation, polished with a 0.5-μm diamond grit grinding disk and subsequently etched by soaking into 20% H_3_PO_4_ for 2 min, were taken by means of scanning electron microscopy (SEM) (Supra 40VP, Zeiss, Oberkochen, Germany). Prior to the analysis, the samples were coated with an approximately 2 nm Au–Pd layer by sputtering.

### Crystallization

The second heat treatments took place in an oven of the Programat^®^ type (Ivoclar Vivadent AG, Schaan, Liechtenstein). Small glass plates of approximately 2 mm thickness, cut from the nucleated glass blocks, were used. A detailed investigation of the temperature-dependent crystal phase formation was conducted considering glass D. The nucleated glass plates were heat treated for 30 min at 520, 540, 560, 580, 600, 700, or 800°C. After the dwell time, the samples were allowed to cool to 500°C in the furnace before opening it. Prior to XRD analysis, conducted as described above, the surface of the glasses and glass–ceramics was removed using a 40-μm diamond grit grinding disk.

A quantitative study of the crystal phase formation was conducted applying Rietveld refinement. Glass–ceramics nucleated at 500°C for 30 min and subsequently crystallized via a second heat treatment for 30 min at 800°C were used. The glass–ceramic material was crushed and comminuted in a mortar grinder (Mortar Grinder RM 200, Retsch, Haan, Germany) and subsequently sieved <45 μm. Then the powder was elutriated with approximately the same mass of Al_2_O_3_ in acetone. The solvent was evaporated in an oven pre-heated at 80°C. Al_2_O_3_ was used as an internal standard in a concentration of about 50 wt.%. After recording the powder XRD patterns from 10° to 100° 2θ in 0.014° steps, the quantification of the crystal phases was done with the TOPAS software from Bruker.

### Microstructure formation

The microstructures of the glass–ceramics crystallized at 800°C for 30 min were investigated applying SEM. Small plates of the nucleated glass blocks were crystallized at 800°C for 30 min and subsequently polished with a 0.5-μm diamond grit grinding disk. The surface of the samples was etched with vapor of 40% HF acid and coated with a 1–2 nm Au–Pd layer prior to SEM analysis. In order utilize the contrast provided by elements with high specific weight via the detection of backscattered ions during SEM, glass–ceramic G was determined. The sample preparation was conducted as described above; however, the surface was etched by soaking into 3% HF acid for 10 s.

### Mechanical and optical properties of the glass–ceramics

Small circular plates were milled from the blocks of nucleated glass using a CEREC^®^ InLab milling machine (Sirona, Bensheim, Germany). Crystallization of the plates took place as described above via a second heat treatment for 30 min at 800°C. The samples were prepared for the testing of the biaxial fracture strength according to ISO 6872 including a surface finishing with a 15 μm diamond grit grinding disk. The measurements were conducted using a universal testing apparatus (Zwick 1456, Zwick GmbH & Co. KG, Ulm, Germany). The biaxial strengths are given as the means of data sets ±SD. Ten samples of each series were tested. Normality of the data sets was determined applying a Jarque–Bera test. Significant deviations (>95%) between the different data sets were examined using the Student’s *t*-test.

Small glass plates measuring approximately 10 mm × 10 mm × 2 mm were cut from the nucleated glass blocks and crystallized at 800°C for 30 min. Afterwards the glass–ceramic plates were prepared for translucency (CR) measurements and color evaluation according to BS5612, DIN5033, and DIN6174, respectively. A spectrometer of the type CM-3700d (Konica-Minolta, Tokyo, Japan) was used for the spectroscopic analysis.

The opalescence *O* of the glass–ceramics was estimated using an equation derived by Kobashigawa et al. (2001) (Eq. 1) and repeatedly used by Lee (Lee et al., 2005, 2006; Lee, 2007), with *a* and *b* being color-related parameters detected with the CM-5 spectrometer (Konica-Minolta, Tokyo, Japan). While the *a*-value gives a measure for the intensity proportion of red and green, the *b*-value deals with yellow and blue. The Δ values *a* and *b* describe the differences of these parameters detected in either the transmission or reflection mode. Glass–ceramic plates measuring approximately 10 mm × 10 mm × 1 mm were used.

(1) $$O=\sqrt{{\left(\Delta a\right)}^{2}+{\left(\Delta b\right)}^{2}}$$

## Results

### Glass formation and thermal analysis

**Figure 1:**
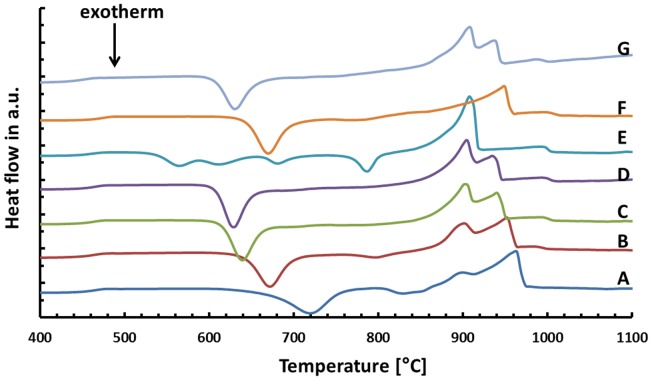
**Thermal analysis of different base glasses**.

The results of the thermal analysis of the base glasses after quenching in water are shown in Figure 1. The exact values for exothermic *T*_exo_ or endothermic *T*_endo_ peak temperatures are given in Table 2. The onset indicating that the glass transition temperature *T*_g_ is in the range of 450–455°C for samples A–E. A slightly increased *T*_g_ can be observed for the F^−^ free reference sample F at about 462°C, while the substitution of CaO by SrO in glass G yields a reduction of *T*_g_ to about 442°C. All samples show at least one exothermic peak in the DSC curve indicating crystallization. Increasing the content of the components CaO, P_2_O_5_, and F^−^ (samples A–E) clearly decreases the temperature for the first exothermic processes detectable by DSC from about 720 to 565°C. A second exothermic signal indicating crystallization is evident at about 830°C for glass A and at approximately 800°C for glass B. However, the latter is rather weak. While glasses C and D as well as F and G do not show any further exothermic signals during thermal analysis, three more exothermic peaks are evident for glass E at about 610, 680, and 786°C.

**Table 2:** **Thermal analysis of the base glasses by means of DSC**.

Base glass		A	B	C	D	E	F	G
*T*_g_	[°C]	451	454	452	457	451	462	442
*T*_exo_1	[°C]	719	672	639	629	564	670	630
*T*_exo_2	[°C]	829	979	−	−	611	772	−
*T*_exo_3	[°C]	849	−	−	−	681	−	−
*T*_exo_4	[°C]	−	−	−	−	786	−	−
*T*_endo_1	[°C]	905	902	904	905	909	949	908
*T*_endo_2	[°C]	963	953	941	937	−	−	936

A comparison of the endothermic signals, which indicates the dissolution of the crystal phases, reveals that no significant difference can be observed between the glasses having different CaO/SrO, P_2_O_5_, and F^–^ contents, with the exception of glass E. All of them show two endothermic peaks between 900 and 965°C. The thermal analysis of the F^–^ free reference sample F reveals only one endothermic signal at about 949°C.

### Nucleation

**Figure 2:**
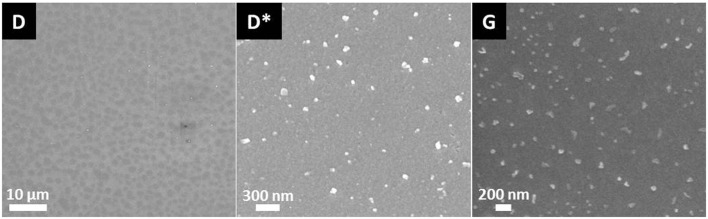
**Microstructures of glass D after nucleation for 10 h at 500°C and of glass G after nucleation at 500°C for 30 min**.

Two different phase separation phenomena could be observed by means of SEM of the glasses after etching the surface with H_3_PO_4_. The investigation of the microstructures revealed cloudy separation areas in the range of 1 μm in diameter as well as sharp non-spherical phase separations with dimensions <100 nm (Figure 2). No crystalline phase formation could be detected by means of XRD analysis of the glass samples after the first heat treatment at 500°C for 30 min or 10 h.

### Crystallization

**Figure 3:**
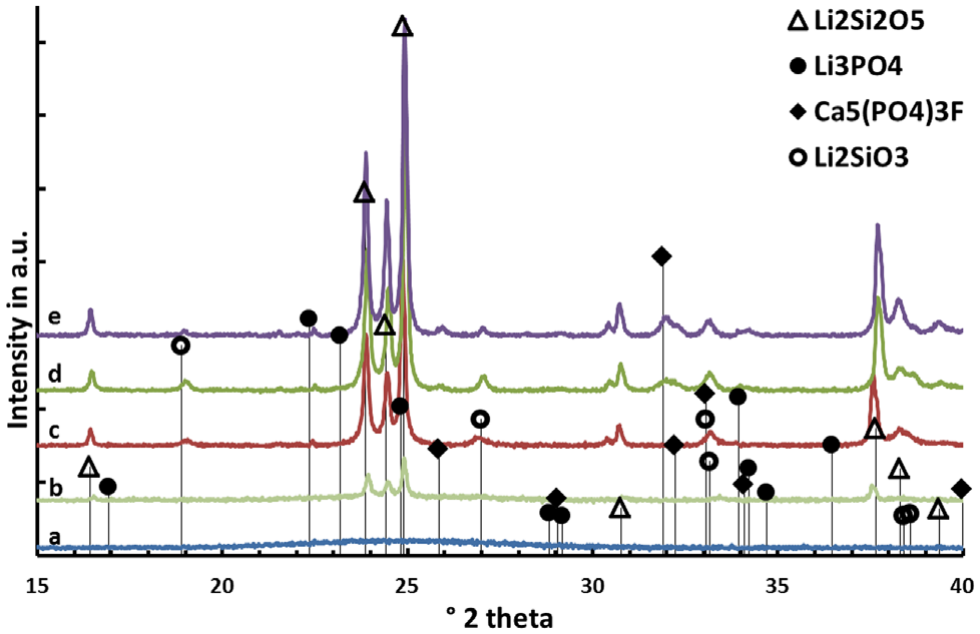
**Diffractograms of glass D after (a) nucleation at 500°C for 30 min and after additional heat treatments at (b) 540°C, (c) 600°C, (d) 700°C, and (e) 800°C for 30 min each**.

Results on the temperature-dependent crystal phase formation in the nucleated base glass D are presented in Figure 3. The nucleated base glass is X-ray-amorphous Li_2_Si_2_O_5_ and traces of Li_2_SiO_3_ crystals are the only crystal phases present in the diffractogram after a second heat treatment at 540°C for 30 min. The precipitation of fluoroapatite can be detected after heat treatments at 700 as well as 800°C. While the relative intensity of the Li_2_SiO_3_ peaks decreases when the crystallization temperature is increased from 700 to 800°C, the contrary can be observed for peaks assigned to fluoroapatite. The presence of crystalline Li_3_PO_4_ was definitely detected after heat treatment at 600°C.

The qualitative and quantitative composition of the glass–ceramics after crystallization at 800°C for 30 min is shown in Table 3. Increasing the content of CaO, P_2_O_5_, and F^−^ in samples A–E enhances the crystallization of fluoroapatite in the glass–ceramics. In the same manner, the amount of residual glass phase is reduced from sample A–D. Glass–ceramic A, B, C, and D contain 56.4, 57.8, 58.7, and 58.1 wt.% Li_2_Si_2_O_5_. Sample E, comprising the largest amount of CaO, P_2_O_5_, and F^−^ and the lowest fraction of Li_2_O and SiO_2_ has the least percentage of Li_2_Si_2_O_5_ (50.3 wt.%) and the highest fraction of Li_2_SiO_3_ (2.9 wt.%) of all the glass–ceramics analyzed. The F^–^ free reference sample F shows the largest content of Li_3_PO_4_ crystals. Compared to the corresponding F^–^ containing glass C, less Li_2_Si_2_O_5_ crystal content is formed. The substitution of CaO by SrO yields the crystallization of Sr_5_(PO_4_)_3_F in glass G.

**Table 3:** **Qualitative and quantitative composition of glass–ceramics after subsequent heat treatment at 500 and 800°C for a duration of 30 min each**.

	A	B	C	D	E	F	G
wt.% Ca_5_/Sr_5_(PO_4_)_3_F	2.0	4.1	6.6	7.4	12.2	–	9.5
wt.% Li_2_Si_2_O_5_	56.4	57.8	58.7	58.1	50.3	54.3	53.6
wt.% Li_3_PO_4_	2.7	1.8	1.9	2.0	1.3	3.5	1.6
wt.% Li_2_SiO_3_	1.5	–	–	–	2.9	–	1.1
wt.% amorphous phase	37.4	36.3	32.8	32.5	33.3	42.2	34.2

### Microstructure formation

**Figure 4:**
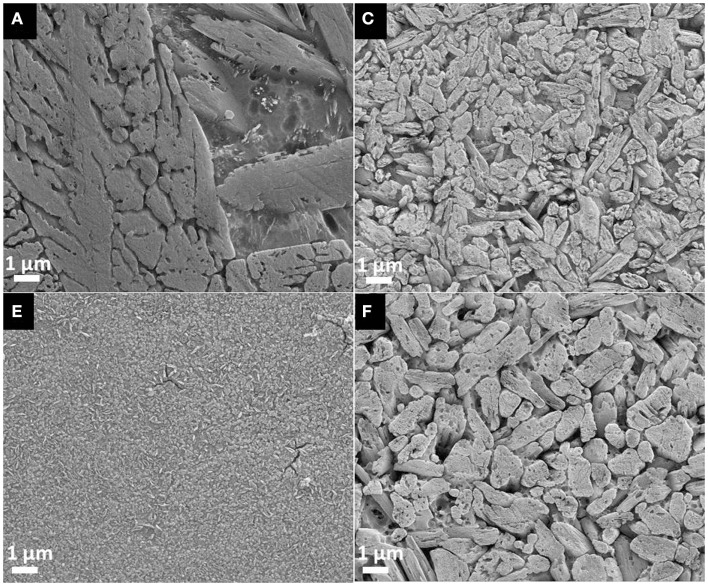
**Microstructure of glass–ceramics A, C, E, and F crystallized for 30 min at 800°C, polished and subsequently etched in 40% HF vapor for 30 s**.

After etching of the polished surface of the glass–ceramics crystallized at 800°C for 30 min with HF vapor, only one type of crystal morphology is evident in each of the micrographs presented in Figure 4. It involves Li_2_Si_2_O_5_ crystals. While the microstructures of glass–ceramics F and G are rather similar, quite different microstructures were found in the glass ceramics A, C, E, and F as shown in Figure 4. Li_2_Si_2_O_5_ crystals in the range of >>10 μm are present in the material A. The size of the Li_2_Si_2_O_5_ crystals clearly decreases with an increasing fraction of CaO, P_2_O_5_, and F^−^, as shown in a comparison of A, C, and E. Furthermore, the presence of F^−^ refines the microstructure as can be seen by comparing glass ceramics C and F.

**Figure 5:**
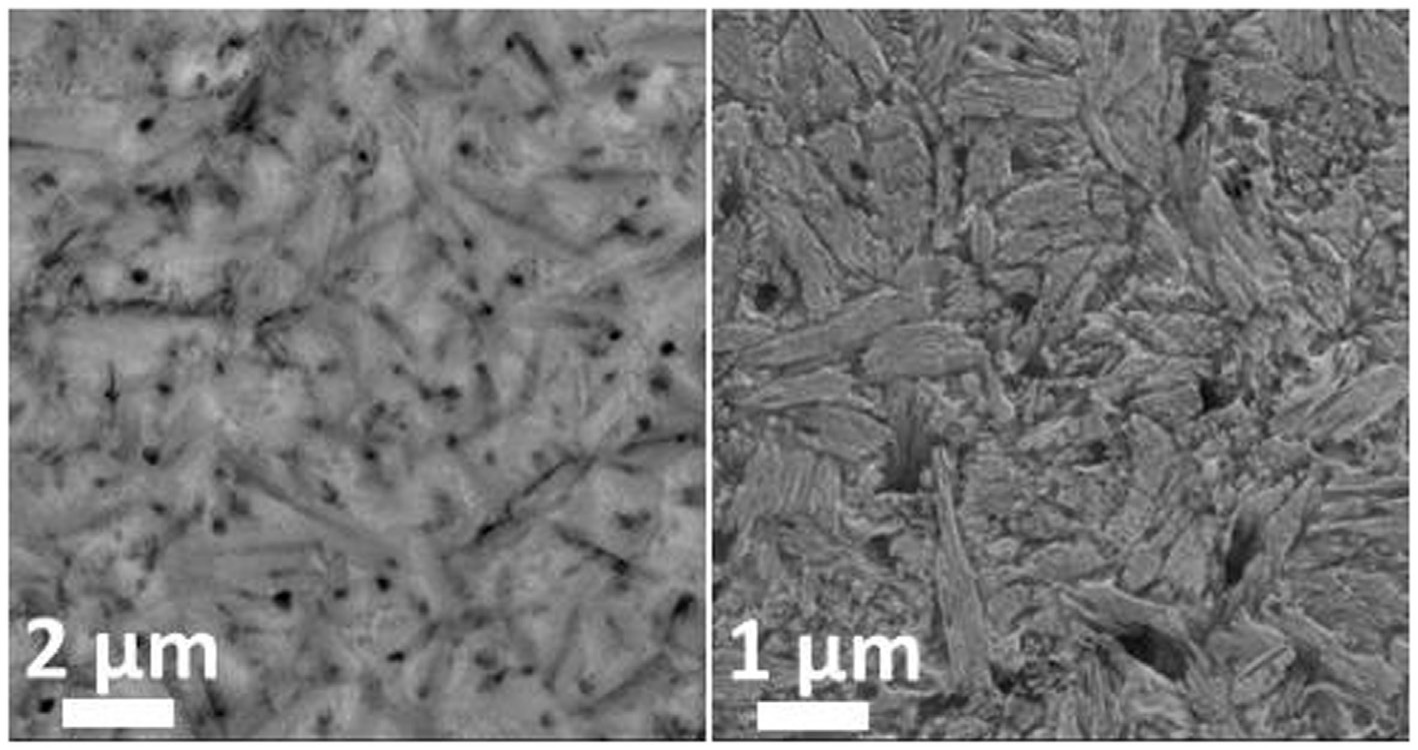
**Microstructure of glass-ceramic G**. Left: backscattered electrons; right: secondary electrons.

The micrographs in Figure 5 display lath or plate-like crystals in a glassy matrix. According to the micrograph taken by backscattered electrons (Figure 5 left), the glass seems to be enriched with ions of relatively high specific weight, such as Sr^2+^, compared to the crystalline structures which obviously comprise rather light ions, such as Li^+^. Regularly distributed black spots with a diameter of approximately 200 nm are present in both micrographs in Figure 5. These black spots indicate holes in the microstructure which are the result of removing a crystalline or amorphous phase from the microstructure by etching.

### Mechanical properties

The biaxial strength of the glass ceramics is presented in Table 4. Glass–ceramic D produces the highest mean strength among all the samples reaching a strength of 538 ± 59 MPa. The strength of sample series D is statistically significantly higher compared to that of the glass–ceramics of types A, B, and E revealing a strength of 217 ± 5, 437 ± 20, and 351 ± 49 MPa. Series A exhibits a significantly lower fracture strength than any other glass–ceramic investigated during this study. The difference in the strength of the materials C, D, F, and G is statistically not significant.

**Table 4:** **Mechanical and optical properties of the glass–ceramics**.

	A	B	C	D	E	F	G
σ_biax_ [MPa]	217 ± 5	437 ± 20	429 ± 103	538 ± 59	351 ± 49	499 ± 34	534 ± 52
CR in%	74.6	69.8	66.1	66.7	50.5	60.3	69.9
O in%	4.5	6.4	9.9	15.2	26.8	11.0	7.2

### Optical properties

The translucency and opalescence properties of the glass–ceramics are presented in Table 4. Increasing the content of CaO, P_2_O_5_, and F^−^ in the compositions A–C decreases the CR value and, hence, reduces the translucency of the glass–ceramics. The relatively small change of the base glass composition from C to D yields a slight increase of the translucency. Composition E shows the highest CR value of all the series. The F^–^ free reference sample F shows a lower translucency compared to that of the fluoroapatite-containing glass–ceramics C and D, while the SrO-containing glass–ceramic G has a slightly decreased translucency compared to the corresponding CaO-containing sample C.

The opalescence of the glass–ceramics increases consistently with an increasing ratio of CaO, P_2_O_5_, and F^−^ in the base glass. There is no significant difference in terms of the apatite-free reference sample F. Only a slightly increased opalescence can be observed compared to that of the corresponding apatite-forming composition C.

## Discussion

Different base glasses, which allow the controlled volume crystallization of Li_2_Si_2_O_5_ as main crystal phase and Ca_5_(PO_4_)_3_F or Sr_5_(PO_4_)_3_F as minor crystal phase, have been developed. In a systematic series of experiments, the components CaO, P_2_O_5_, and F^−^ were simultaneously increased from base glass A to base glass E in the stoichiometric ratio of Ca_5_(PO_4_)_3_F. The increase of the mentioned components was conducted at the expense of SiO_2_ and Li_2_O, ensuring a constant molar ratio of SiO_2_/Li_2_O. Besides the components, necessary for the formation of the desired crystal phases, the glass system was extended by Al_2_O_3_ and K_2_O in order to enhance the processability and chemical stability of the glasses.

**Figure 6:**
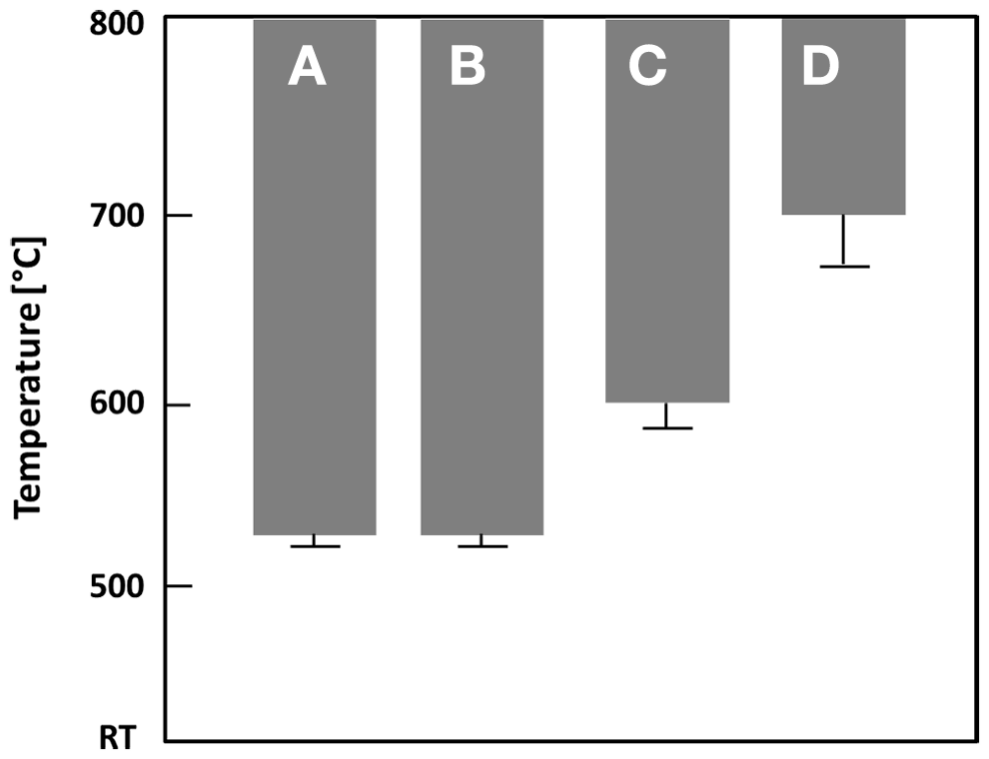
**Schematic summary of the phase formation sequence of Li_2_Si_2_O_5_ (A), Li_2_SiO_3_ (B), Li_3_PO_4_ (C), and Ca_5_(PO_4_)_3_F (D) as a function of crystallization temperature for glass D after nucleation at 500°C for 30 min**.

Beyond controversy, the P_2_O_5_ content plays an important role in the studied glass system. It is essential for the nucleation of lithium silicate as well as mandatory for the crystallization of Ca_5_(PO_4_)_3_F. The preliminary study for nucleation by means of SEM (Figure 2) of the base glasses after heat treatment at 500°C for 30 min revealed the presence of phase separations. There is no crystalline phase present according to XRD analysis (Figure 3), though. The shape and size of the phase separations visible in the micrographs in Figure 2 (D* and G) indicate a nanocrystalline phase, which is observable after etching with H_3_PO_4_. The phase separations being amorphous or disordered nanocrystalline phases of the Li_3_PO_4_ kind seems likely according to previous observations made in non-stoichiometric Li_2_Si_2_O_5_ forming glasses (Bischoff et al., 2011). On a larger scale (> 1 μm), there were rather diffuse separation areas visible (Figure 2, D) which give evidence for the enrichment of ions, most probably P_2_O_5_, in certain areas. Due to the huge discrepancy in the size of the two different kinds of phase separation phenomena observed, they seem to be independent of each other. Assuming that both are enriched with P_2_O_5_, since etching with H_3_PO_4_ uncovered the phases, the formation of P_2_O_5_ sites enriched with Li^+^, on the one hand, as well as the parallel formation of P_2_O_5_ sites enriched with Ca^2+^ ions should be considered. While the Li^+^-rich sites could be responsible for the formation of small amorphous or disordered nanocrystalline Li_3_PO_4_ phase separations, known to nucleate lithium silicate (Höland and Beall, 2012), the large and rather cloudlike areas could accommodate Ca^2+^ rich P_2_O_5_ sites which later on initiate apatite formation. Depletion of these glass areas of Li^+^ and Si^4+^-Q-groups coordinated in tetrahedrons with O^2−^, as a consequence of lithium silicate precipitation, could have an enhancing effect on the crystallization of apatite, which occurred clearly subsequently to the early formation of lithium silicate and Li_3_PO_4_ (Figures 3 and 6).

The principles of twofold crystallization of Li_2_Si_2_O_5_ and Ca_5_(PO_4_)_3_F in glass D, based on the XRD patterns shown in Figure 3, are summarized in the schema presented in Figure 6. As a function of temperature, Li_2_Si_2_O_5_ and Li_2_SiO_3_ are the first two crystalline phases that formed at temperatures above 540°C, prior to the precipitation of Li_3_PO_4_ at temperatures <600°C. The formation of crystalline Li_3_PO_4_ occurred surprisingly about 180°C below the temperatures reported so far in the literature for P_2_O_5_ containing Li_2_Si_2_O_5_ glass systems (Höland and Beall, 2012). P_2_O_5_ phase separation phenomena and the early parallel formation of Li_2_Si_2_O_5_ and Li_2_SiO_3_ happen in analogy to the principles reported in the literature (Höland and Beall, 2012), which describe the nucleating effect of amorphous or disordered nanocrystalline Li_3_PO_4_ phases on these crystal phases. The Ca_5_(PO_4_)_3_F crystal phase formation happening at temperatures definitely above the crystallization of lithium silicates as well as Li_3_PO_4_ seems to be independent and subordinated, though. However, comparing the crystal phase compositions of the apatite-forming glass–ceramic C with the corresponding F^−^ free reference sample F (Table 3), one can see the significantly increased Li_3_PO_4_ content in the apatite-free material F. The structural integration of P_2_O_5_ in, e.g., an amorphous fluoroapatite pre-phase and, hence, hindering the crystallization of Li_3_PO_4_ could explain this phenomenon. Further in-depth investigation of the involved mechanism is necessary to identify the exact mechanisms running.

**Figure 7:**
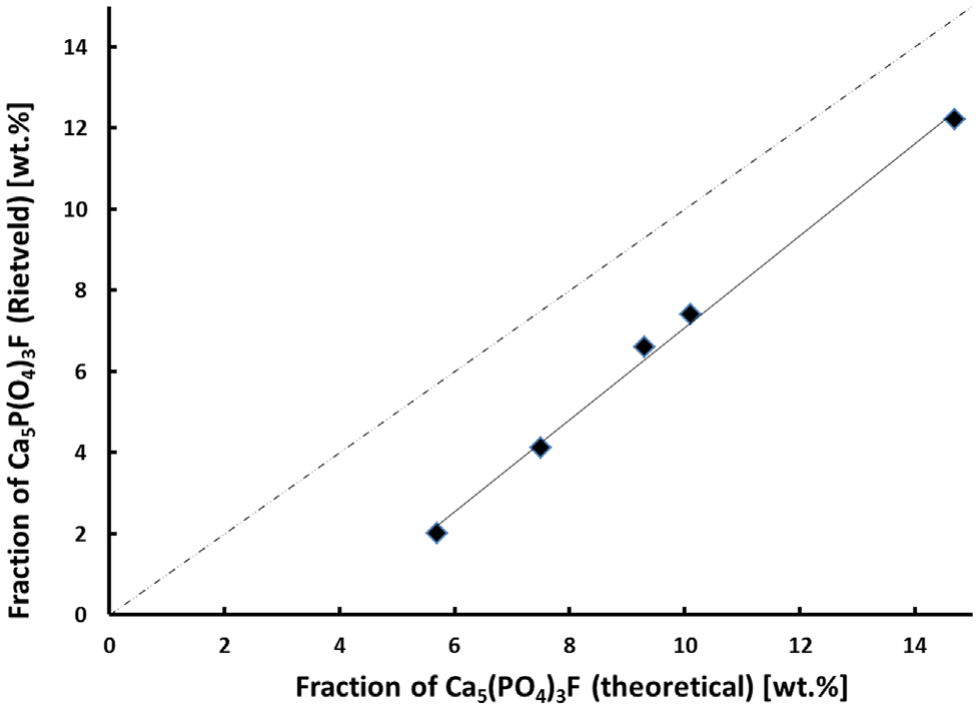
**Experimentally estimated fraction of Ca_5_(PO_4_)_3_F as a function of the theoretically feasible fraction based on the chemical glass composition**.

Although the exact crystallization and nucleation mechanisms are still a point of discussion, the qualitative and quantitative crystallization of fluoroapatite as minor phase in Li_2_Si_2_O_5_-dominated glass–ceramics could be precisely controlled via the chemical composition of the base glasses (Table 3). Lithium disilicate and fluoroapatite could be precipitated in bulk glasses by means of twofold crystallization. The content of fluoroapatite in the glass–ceramic materials could be controlled by raising the ratio of apatite-forming oxides and ions CaO, P_2_O_5_, and F^−^ in a lithium disilicate forming glass composition (Tables 1 and 3). Assuming, for the sake of simplicity, that fluoroapatite forms prior to and independently of the formation of Li_2_Si_2_O_5_ and Li_3_PO_4_, the maximum theoretically possible content of fluoroapatite in the glass–ceramics can be calculated. Figure 7 shows the actual percentage of Ca_5_(PO_4_)_3_F detected and estimated by means of powder XRD in combination with Rietveld refinement as a function of the theoretically possible fraction based on the chemical composition of the glasses. The content of fluoroapatite increases linearly with the increasing ratio of apatite-forming components. However, according to the slope of the linear regression curve in Figure 7, the crystallization of fluoroapatite becomes more efficient with the increasing content of those components, since the difference between the theoretically estimated and the experimentally determined values decreases from approximately 3.7 to 2.5 wt.%. Similar to the hindering effect of F^−^ on the crystallization of Li_3_PO_4_, discussed above, the latter could be explained by the formation of amorphous CaO-P_2_O_5_-F rich phase separation sites within the glass microstructure. This phenomenon was already reported in the literature (Höland and Beall, 2012). Furthermore, a higher content of F^−^, which acts as network modifier, increases the number of disconnecting points in the glass network (Vogel, 1994) and, thus, facilitates required diffusion processes. With regard to the sample series C and F, the significant shift of the first exothermal DSC signal to a lower temperature resulting from the addition of F^−^ could be further evidence for the enhancing influence of F^−^ on crystallization. Quasi isothermal crystallization or long-term crystallization experiments could help to gain valuable information on this topic.

**Figure 8:**
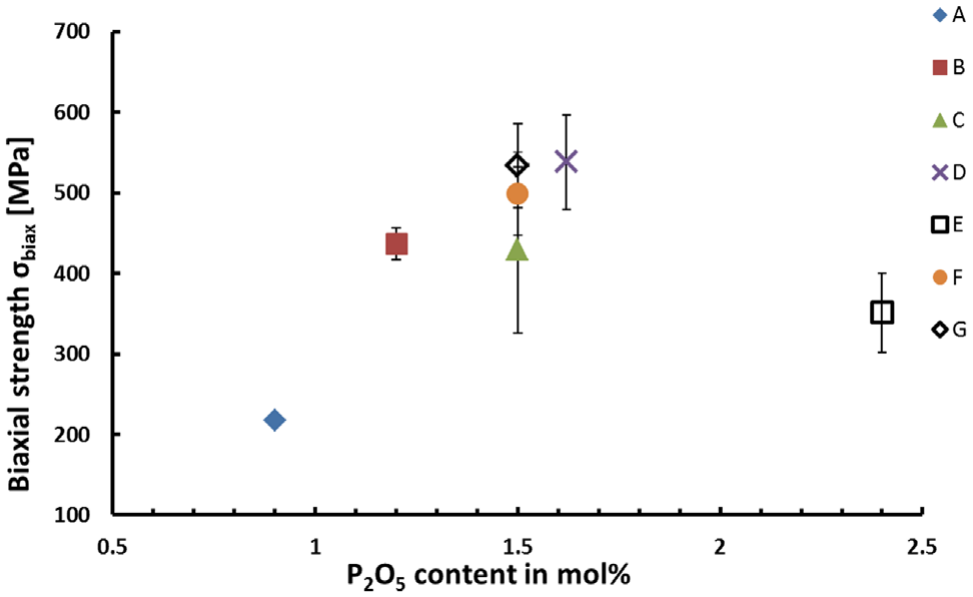
**Biaxial strength as a function of the P_2_O_5_ content of the glass-ceramics nucleated at 500°C for 30 min and subsequently crystallized at 800°C for 30 min**.

High biaxial strengths of >500 MPa could be achieved for glass–ceramics containing more than 5 wt.% apatite. The experimental results prove that apatite could be precipitated as minor crystal phase in glass–ceramics without affecting the mechanical properties of these materials. The micrographs shown in Figure 5 provide an indirect proof for the existence of a most probably needle-like, nanometer-scaled apatite phase randomly distributed within the Li_2_Si_2_O_5_ microstructure. However, suited sample preparation parameters for the direct observation of apatite crystals in the present glass–ceramics have not yet been found. Nevertheless, the microstructural effects of Li_2_Si_2_O_5_ crystals dominate the mechanical properties. The influence of their size and morphology on the strength of glass–ceramics have been established by various authors in a multitude of investigations (Cramer von Clausbruch et al., 2000; Höland et al., 2005; Apel et al., 2007, 2008; Zheng et al., 2008; Dittmer et al., 2014). In summary, a high volume fraction of interlocking lath-like Li_2_Si_2_O_5_ crystals yields high-strength materials. The most efficient way of controlling the microstructure of lithium disilicate glass–ceramics derived from bulk glasses is by means of internal nucleation sites, in most cases via the content of P_2_O_5_ (Cramer von Clausbruch et al., 2000; Höland et al., 2005). Similar to the results of numerous studies presented so far in the systems SiO_2_–Li_2_O–Al_2_O_3_–K_2_O–(ZrO_2_)–P_2_O_5_ (Höland et al., 2006; Höland and Beall, 2012), the findings of the present work clearly suggest the nucleating effect of the P_2_O_5_ content on the microstructure (Figure 4) and the biaxial strength as demonstrated in Figure 8. According to the mean values, there seems to be an optimum with respect to the biaxial strength which should be at approximately 1.6 mol% P_2_O_5_. As presented in Figure 4, C, interlocking plate-like crystals in the range of approximately 1 μm yield the best results. Obviously, the microstructures of A (Figure 4, A) and E (Figure 4, E) were too coarse or too fine, respectively, for imparting good mechanical strengths to the glass–ceramic material.

Similar to the mechanical properties described above, the optical properties were mostly influenced by the morphology and size of Li_2_Si_2_O_5_ crystals in the microstructure. Major effects on the translucency and opalescence can rather be correlated to the P_2_O_5_ content and, hence, the morphology and size of Li_2_Si_2_O_5_ crystals, than to the content of fluoroapatite in the glass–ceramic. However, a study of the translucency of the Ca_5_(PO_4_)_3_F-comprising glass–ceramic C and the corresponding apatite-free material F revealed a certain effect of the apatite crystals on this optical property. Although the Li_2_Si_2_O_5_ crystals visible in the micrograph of glass–ceramic F (Figure 4, F) are significantly larger than the ones found in C (Figure 4, C), glass–ceramic F exhibits a better translucency than C. This is in contrast to the literature and the knowledge about glass–ceramics and ceramics (Höland and Beall, 2012). One would expect to obtain improved translucency with finer microstructures, since scattering effects at interfaces or grain boundaries will decrease with decreasing sizes (Nassau, 2001). This effect appears especially for crystal sizes near or even below the wavelength spectrum of visible light and, hence, provides evidence that submicron-sized apatite crystals distributed within the Li_2_Si_2_O_5_-dominated microstructure introduce a further type of interface besides the one formed by Li_2_Si_2_O_5_ as well as Li_3_PO_4_ crystals and the glass matrix. Optical reflection and scattering due to this additional type of phase boundary could explain the relatively high opacity of glass–ceramic C compared to that of F.

In summary and with respect to crystallization phenomena, the twofold precipitation of Li_2_Si_2_O_5_ and (Ca/Sr)_5_(PO_4_)_3_F provides a further means of precisely controlling the optical properties of lithium disilicate glass–ceramics, which is a prerequisite for the successful application of this material in prosthodontics. Of major importance is the fact that the precipitation of the minor phase can be specifically controlled without affecting the good mechanical and chemical properties of Li_2_Si_2_O5 glass–ceramics, which are relevant to dental applications. Investigations that have been running in parallel to the present study have proved this to be true for the fracture toughness as well as the chemical durability.

## Conclusion

The controlled precipitation of (Ca/Sr)_5_(PO_4_)_3_F as a minor phase in Li_2_Si_2_O_5_-based glass–ceramics from bulk glasses via a solid-state reaction can be achieved by adding CaO/SrO, P_2_O_5_, and F^−^ in multi-component base glasses.

The investigation of two different phase separation phenomena in the nucleated base glasses indicates the existence of chemically different P_2_O_5_ sites.

A strong nucleating activity of amorphous or disordered nanocrystalline P_2_O_5_ on the crystallization of lithium silicate could be approved, while there is no evidence for the need of any crystalline heterogeneous phase for the nucleation of fluoroapatite. Lithium silicate crystal phase formation starts ca. 160°C prior to the crystallization of Ca_5_(PO_4_)_3_F. The authors conclude on the existence of two different solid-state reactions without competitive character.

The quantity of apatite precipitated in the glass–ceramics is almost directly proportional to the content of apatite-forming components provided in the base glasses. The efficiency of apatite crystallization increases with the increasing content of CaO/SrO, P_2_O_5_, and F^−^.

The mechanical and optical properties of the high-strength and chemically durable glass–ceramics are for the most part influenced by the morphology and size of the Li_2_Si_2_O_5_ crystals. However, the precipitation of Ca_5_(PO_4_)_3_F or Sr_5_(PO_4_)_3_F facilitates the precise control of the translucency and opalescence which is a prerequisite for the successful application of the material in restorative dentistry.

## Conflict of Interest Statement

The authors are employees of the company Ivoclar Vivadent AG. A patent was filed on the present subject.
